# Detection of QTL with effects on osmoregulation capacities in the rainbow trout (*Oncorhynchus mykiss*)

**DOI:** 10.1186/1471-2156-12-46

**Published:** 2011-05-14

**Authors:** Yvan Le Bras, Nicolas Dechamp, Francine Krieg, Olivier Filangi, René Guyomard, Mekki Boussaha, Henk Bovenhuis, Thomas G Pottinger, Patrick Prunet, Pascale Le Roy, Edwige Quillet

**Affiliations:** 1 INRA, UR1037 SCRIBE, IFR 140, F-35000 Rennes, France; 2 INRA, UMR1313 Génétique Animale et Biologie Intégrative, F-78350 Jouy-en-Josas, France; 3 Animal Breeding and Genetics Group, Wageningen University, P.O. Box 338, NL-6700AH, Wageningen, The Netherlands; 4 Centre for Ecology & Hydrology, Lancaster Environment Centre, Bailrigg, Lancaster LA1 4AP, UK; 5 INRA, UMR0598 Génétique Animale, F-35000 Rennes, France; 6 Agrocampus Ouest, UMR0598 Génétique Animale, F-35000 Rennes, France

## Abstract

**Background:**

There is increasing evidence that the ability to adapt to seawater in teleost fish is modulated by genetic factors. Most studies have involved the comparison of species or strains and little is known about the genetic architecture of the trait. To address this question, we searched for QTL affecting osmoregulation capacities after transfer to saline water in a nonmigratory captive-bred population of rainbow trout.

**Results:**

A QTL design (5 full-sib families, about 200 F2 progeny each) was produced from a cross between F0 grand-parents previously selected during two generations for a high or a low cortisol response after a standardized confinement stress. When fish were about 18 months old (near 204 g body weight), individual progeny were submitted to two successive hyper-osmotic challenges (30 ppt salinity) 14 days apart. Plasma chloride and sodium concentrations were recorded 24 h after each transfer. After the second challenge, fish were sacrificed and a gill index (weight of total gill arches corrected for body weight) was recorded. The genome scan was performed with 196 microsatellites and 85 SNP markers. Unitrait and multiple-trait QTL analyses were carried out on the whole dataset (5 families) through interval mapping methods with the QTLMap software. For post-challenge plasma ion concentrations, significant QTL (P < 0.05) were found on six different linkage groups and highly suggestive ones (P < 0.10) on two additional linkage groups. Most QTL affected concentrations of both chloride and sodium during both challenges, but some were specific to either chloride (2 QTL) or sodium (1 QTL) concentrations. Six QTL (4 significant, 2 suggestive) affecting gill index were discovered. Two were specific to the trait, while the others were also identified as QTL for post-challenge ion concentrations. Altogether, allelic effects were consistent for QTL affecting chloride and sodium concentrations but inconsistent for QTL affecting ion concentrations and gill morphology. There was no systematic lineage effect (grand-parental origin of QTL alleles) on the recorded traits.

**Conclusions:**

For the first time, genomic loci associated with effects on major physiological components of osmotic adaptation to seawater in a nonmigratory fish were revealed. The results pave the way for further deciphering of the complex regulatory mechanisms underlying seawater adaptation and genes involved in osmoregulatory physiology in rainbow trout and other euryhaline fishes.

## Background

The expansion of intensive aquaculture for salmonids in seawater net pens in Europe and western North America has heightened interest in genetic studies of a number of production traits in these species. Particularly well studied are aquacultured species such as rainbow trout (*Oncorhynchus mykiss*) which, in their wild state, include both migratory (steelhead trout) and nonmigratory (rainbow trout) forms.

Various studies have shown that rainbow trout reared in the marine environment (estuarine or coastal areas) show better growth compared to fish reared in freshwater [1-3]. Strains that naturally smoltify, a complex physiological and behavioural change that pre-adapts the fish to a high salinity environment, can be used for such purposes. However, nonmigratory populations can also be successfully adapted to, and reared in, seawater.

The ability of rainbow trout to adapt to seawater (SW) depends on the development of branchial ionoregulatory mechanisms. In a hyperosmotic environment (SW), teleost fish lose water through osmosis and gain ions (essentially Na^+ ^and Cl^-^) through diffusion. Maintenance of a stable hydromineral balance mainly relies on ingestion of SW coupled with active excretion of Na^+ ^and Cl^-^. In freshwater (FW), the reverse mechanisms occur (see review by Evans *et al*. [4]). Numerous studies have investigated the activity of ion-transporters during acclimation to SW, particularly at the gill epithelium where Na^+ ^and Cl^- ^are actively excreted in order to regulate plasma Na^+ ^and Cl^- ^levels. These effluxes rely on branchial epithelial transporters such as Na^+^/K^+^-ATPase and Na^+^-K^+^-2Cl^- ^for Na^+ ^and CFTR for Cl^- ^(see reviews Evans *et al*. [4]; Hwang and Lee [5]).

Several studies have clearly shown that the size of the fish and conditions of transfer (for example the salinity gradient) from FW to SW are key parameters for successful adaptation of rainbow trout to hyperosmotic environment [3,6]. Genetic factors can also contribute to the ability of fish to adapt to SW environment. Differences between two reciprocal interspecific hybrid bass populations (white bass *Morone chrysops *x striped bass *Morone saxatilis*) were shown for plasma osmolality during acclimation to salinity [7]. Differences between lagoon and marine sea bass (*Dicentrarchus labrax*) before and after freshwater acclimation have also been reported by Allegrucci *et al*. [8]. The inheritance of smoltification has been examined in several salmonid species including steelhead trout (the migratory form of rainbow trout) [9-11]. These studies suggest that both the timing and propensity to smoltification are under genetic control. Similar findings have been reported for migratory (anadromous) and nonmigratory populations of Atlantic salmon (*Salmo salar*). Surveys of landlocked Atlantic salmon from North America and Europe, and of landlocked and anadromous populations have shown differences in the capacity of these strains to adapt to SW [12-16] and the analysis of osmoregulatory mechanisms at the level of gill has shown that differential expression of gill Na^+^/K^+^-ATPase -α1a, -α1b and -α3 isoforms associated with Na^+^/K^+^-ATPase activity may account for these differences in osmoregulatory performance [17].

The recent development of genetic resources in rainbow trout, including genetic markers and medium density genetic maps [18-21] provides the foundation for deeper biological understanding of the genetic basis of phenotypic patterns of importance for aquaculture. The identification of QTL (Quantitative Trait Loci) paves the way for a precise investigation of the molecular genetic basis of traits. While many QTL studies have already been implemented for a range of traits, including growth [22], disease resistance [23-26], temperature tolerance [27], cortisol responsiveness [28] and early maturation [29], very little has been done regarding osmoregulation capacities in rainbow trout. Nichols *et al*. [10] performed a QTL study for smoltification-related traits using a cross between nonmigratory (rainbow) and migratory (steelhead) trout lines. However, the study mainly focused on growth and morphological traits (body shape, skin reflectance) while no significant QTL was detected for the gill Na^+^/K^+^-ATPase activity, a key branchial epithelial Na^+ ^transporter.

In this study, we transferred rainbow trout from FW to SW and performed a QTL analysis for plasma Na^+ ^and Cl^- ^levels, which are the standard physiological parameters used to characterize the ability of fish to adapt to a hyperosmotic environment [30-32]. In addition, we were also interested in gill size which could be a factor in salinity adaptation. A QTL design was produced using rainbow trout lines previously selected for high (HR) or low (LR) cortisol responsiveness to an acute confinement stressor [33]. Cortisol is a major hormone regulating stress responses but also a major osmoregulatory hormone. Though mechanisms induced by confinement and salinity stressors may not be the same, the HR and LR lines differ in the responsiveness of the corticotrope axis and thus represented highly relevant biological material for this QTL experimental design.

## Methods

### Experimental design and fish rearing conditions

Grand-parental brood stock belonged to two *O. mykiss *lines (HR and LR), previously selected for divergent plasma cortisol responsiveness to a standardized confinement stressor [33,34]. F1 parents were produced by mating single individuals within the second generation of selected HR and LR individuals, one from each line. The next generation, five F1 males and five F1 females were single pair mated in order to produce the five F2 full sib families used for QTL detection (denoted X3, X4, X8, X14 and X17). All fish (F0, F1 and F2 families) were maintained at the CEH (Centre for Ecology & Hydrology) experimental fish facilities (Windermere, UK).

Fertilization was performed in January, 2006, employing standard stripping and fertilization methods. When the fish were about 11 months old, 215 individuals per family were randomly sampled, individually tagged with passive integrated transponders (PIT; Trovan ID100A), fin clipped for further DNA extraction and distributed into ten holding tanks (1000 litres), with each family held in two tanks. Each tank was supplied with a constant flow of lake water (25 L/min) at ambient temperature. The fish were monitored closely and fed approx 2% body weight, 3 days per week (Skretting Standard Expanded 40). As part of the EU Aquafirst project, fish were submitted to two successive confinement stressors when they were about 15 months old. At the end of the confinement challenge, each family was distributed into holding tanks at a similar density (around 40 to 50 fish per tank, *i.e. *5 to 6 tanks per family depending on the family size). Fish were allowed to recover until the commencement of the osmotic challenge (18 months old).

The experimental work at CEH Windermere was carried out under the UK Animals (Scientific Procedures) Act 1986, Project Licence no. 40/2600.

### Osmotic challenge and measurements

Each fish was tested twice with 14 days between challenges. Exposure to salt water was carried out in a series of 20 plastic tanks that were filled with 150 litres of lake water containing 4500 g Red Sea Coral Reef Salt (30 ppt) and continuously aerated and fitted with an opaque lid. The contents of a single holding tank (40 to 50 fish) were accommodated in four salinity test tanks allowing the contents of four holding tanks to be tested on each working day. The fish were transferred on day 1 and, as osmotic stress acclimation is a temporal process, they were sampled exactly 24 h later, on day 2, corresponding to the time required for the maximum acclimatory response. Fish were netted from the salinity test tanks and anaesthetized in buckets containing 2-phenoxyethanol (1:2000) before being blood sampled and identified by PIT tag. Blood was collected from the Cuverian sinus into heparinised syringes and plasma was stored frozen until required for determination of plasma chloride and sodium concentrations. Fish were returned to their holding tanks to recover. During the first test, body weight and fork length were recorded for each fish.

Plasma sodium and chloride levels were measured by flame photometry (VWR international, Fontenay-sous-bois, France) and by colorimetry (chlorure LDM SOBIODA^®^) [35] respectively. Plasma samples were diluted 1:400 and 1:2 with distilled water for the analysis of sodium and chloride respectively and analyses were performed in duplicate and triplicate respectively. Sodium values from the first and second challenge were identified as Na^+^1 and Na^+^2 respectively, and the corresponding chloride values were denoted Cl^-^1 and Cl^-^2.

Fish were sacrificed about 2 months after the end of the second challenge. At that time, the whole body weight, the weight of gill archs (including gill filaments and cartilage) and sex (macroscopic examination of the gonads) were recorded for the remaining individuals. An index of relative gill weight, named gill index (gill weight corrected for body weight used as covariable) was included in the present study, with the intention of investigating possible links between gill size and osmoregulation capacities. Indeed, a preliminary survey of gill morphology in 40 fish from HR and LR lines revealed that LR individuals had larger gill arches in term of weight and surface area. It was assumed that the individual relative gill weight remains stable across short periods of time and at the time of terminal sampling was still representative of the morphology at the time of osmotic challenge.

### Genotyping and genetic map

The genome scan was performed using microsatellites and SNP markers. Microsatellites were chosen from the INRA reference linkage maps [19]. In a first step, 201 microsatellites chosen on the basis of their map position were screened for polymorphism in the original HR and LR lines, and a sub-set of 138 was retained for further genotyping. In a second step, in order to improve the genome coverage within every family, additional markers were selected in genomic regions still poorly covered and were tested for polymorphism in the five QTL families. In the end, a set of 196 microsatellites, including 13 duplicated markers, was used. Among those, 49 were genotyped by LABOGENA (Jouy-en-Josas, France) while the others were genotyped at INRA-GABI. SNP markers designed by Krieg *et al*. (in preparation) were screened for polymorphism in the QTL families and 85 (see GenBank dbSNP submission numbers in Additional file 1) were retained for the genome scan and genotyped by Genoscope using SNPlex Genotyping System (Applied Biosystem). The overall polymorphism information content (PIC) of markers was 0.51 for microsatellites and 0.30 for SNP. The mean PIC per linkage group ranged from 0.32 to 0.65.

The genetic consensus linkage map was rebuilt for the families of the QTL design including the whole set of markers and using the CarthaGène software ([36], http://www.inra.fr/internet/Departements/MIA/T//CarthaGene/). The total length of the map was 3182 cM, so that the mean overall spacing for genome coverage was about 16 cM (3 to 16 markers per linkage group; 179 to 206 informative markers per family). Linkage groups were named according to Guyomard *et al*. [19], with correspondence with physical chromosomes according to Phillips *et al. *[20] indicated in tables and figures.

### Statistical analyses

#### Description of traits

We first provided some description of the raw data for the five traits of interest (Na^+^1, Na^+^2, Cl^-^1, Cl^-^2 and gill index) and determined whether the rank of challenge (first *vs *second challenge) on one hand and the different families on the other hand significantly affected the traits. The significance of these two factors was tested using the GLM procedure, SAS version 9 [37] at the P < 0.05 level.

#### Sources of variability

In order to choose a model for further analyses, the effects of several fixed effects and co-factors were tested for every trait. The GLM procedure, SAS version 9 [37] was applied to an overall model which included tested factors and the full-sib family effect. For plasma ion values (Na^+^1, Na^+^2, Cl^-^1, Cl^-^2) the overall model was:(1)

where

Y_ijklmn _is the progeny phenotype (plasma ion values)

μ is the general mean of the model

Sex_i _is the fixed effect of sex i with 2 levels (male and female)

Date_j _is the fixed effect of the date j of challenge (6 levels)

Htank(date)_jk _is the fixed effect of the holding tank k within date j (21 levels)

Ctank_l _is the fixed effect of the challenge tank l (20 levels)

Cross_m _is the fixed effect of the full-sib family m (5 levels)

weight is the body weight at the first challenge considered as a covariable

length is the fork length at the first challenge considered as a covariable

and e_ijklmn _is the residual of the model supposed normally distributed with a mean 0 and a variance σ_e_^2^.

For gill weight, the model was adapted to take into account further changes in the management of fish between the end of challenge and measurements of sex and gill weight.(2)

where

Y_ijklm _is the progeny gill weight value

μ is the general mean of the model

Sex_i _is the fixed effect of sex i with 2 levels (male and female)

Group_j _is the fixed effect of the date j at weighing (2 levels, as after challenge 2, fish were redistributed in 2 groups that were terminally sampled with several weeks delay)

Htank_k _if the fixed effect of the holding tank k (12 levels)

Cross_l _is the fixed effect of the full-sib family l (5 levels)

weight is the body weight at the date of measurement considered as a covariable

length is the fork length at the date of measurement considered as a covariable

and e_ijklm _is the residual of the model supposed normally distributed with a mean 0 and a variance σ_e_^2^.

Factors having a P-value equal to or less than 0.2 in these pre-analyses were kept in the final models. Model (1) for ion plasma concentrations revealed that all effects but sex and fork length were significant at the 0.20 level. Model (2) for gill weight indicated that body weight, sex (males having heavier gills than females) and Group were significant at the 0.20 level. Thus, the final models used in further analyses were as follows:

For plasma ion concentrations (both challenges):(3)

For gill index:(4)

#### Phenotypic correlations

Models (3) and (4) were applied to adjust raw data for fixed effects and co-factors using the SAS GLM procedure. The Pearson coefficients of correlation were then determined with the CORR procedure, SAS version 9 [37] applied on residual values of these models (phenotypic correlations).

#### QTL detection

QTLMap software [38] was used for QTL detection, scanning for QTL every 1 cM in the genome. An interval mapping method described by Elsen *et al. *[39] was applied considering a set of non-related full-sib families design and making no assumption about allele numbers or allele frequencies at QTL within founder populations. QTL effects are thus estimated, for each sire and each dam, as the allelic substitution effects. The statistical test used to compare the hypotheses of the presence of one QTL (H1) *vs *no QTL (H0) at one location was an approximate likelihood ratio test (LRT) [40]. The empirical distribution of LRT was obtained from 1000 simulations under the null hypothesis, with a trait heritability fixed to 0.5, for each trait and each linkage group. Thresholds of H0 rejection at the chromosome-wide level (P-values = 0.005, 0.01, 0.05 and 0.10) were then estimated with the method described by Harrel and Davis [41]. A QTL with a P-value < 0.10 at the chromosome-wide level was retained as suggestive, and a QTL with a P-value < 0.05 at the chromosome-wide level was retained as significant [42]. The widely used "one LOD drop-off method" was applied to obtain 95% confidence intervals of the QTL location [43]. Finally, the status of the sires and dams for QTL (heterozygous *vs *homozygous) was tested using a t-test (P < 0.05). As grand-parents had been genotyped, the lineage origin, *i.e*. HR or LR, of each QTL allele of heterozygous parent could be deduced.

In a first step, univariate analyses were carried out trait by trait. Multiple-trait QTL analyses were performed in a second step. We applied a multivariate model with a multinormal penetrance distribution, which is the most powerful and accurate method [44]. In both unitrait and multiple-trait analyses, the phenotypic models (3) and (4) were given as input of the QTLMap software for the analysis of raw data.

## Results

### Traits description

The number of recorded individuals, family means, overall means and standard deviations of the different traits (raw data) are given in Table 1.

**Table 1:** Mean body traits and ion plasma concentrations at the two successive osmotic challenges

Traits	Unit	Family mean					Overall mean	SD	n
					
		X3	X4	X8	X14	X17			
Na^+^1	mmol.L^-1^	185	195	209	186	191	193	27	932
Na^+^2		204	207	221	206	200	208	35	900
Cl^-^1		147	144	156	140	139	145	17	931
Cl^-^2		147	142	141	148	138	143	15	894

Body weight at challenge 1	g	203	200	210	190	214	204	9	932

Fork length at challenge 1	cm	26.6	25.7	26.5	26.2	26.7	26.3	0.4	932

Final gill weight	g	10.8	10	10.8	9.6	11.0	10.4	2.6	787
		
Final body weight		423	382	433	381	434	411	98	791

Na^+^1, Na^+^2: plasma sodium concentration at first and second challenge respectively.

Cl^-^1, Cl^-^2: plasma chloride concentration at first and second challenge respectively.

Final gill weight and body weight measured 2 months after the end of the second challenge.

SD: overall standard deviation; n: total number of individual records.

**Figure 1:**
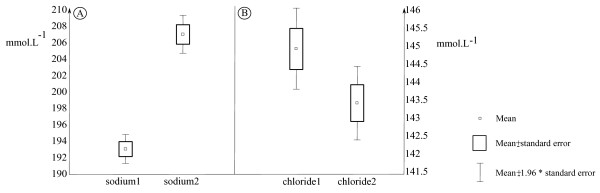
**Boxplot representation of sodium (A) and chloride^- ^(B) plasma concentrations at the two successive osmotic challenges**.

Figure 1 illustrates the effect of the challenge (first *vs *second challenge), with a marked increase of plasma sodium concentrations during the second challenge relative to the first (+8.5% in average, P < 0.01, from +5.7 to +10.7% depending on the family) whereas chloride concentrations were lower during the second challenge (-1.2% in average, P < 0.05, from -10% to +5% depending on the family) than during the first. Significant family differences (P < 0.01) were found only for gill index and Na^+^2. For other plasma ion concentrations (Na^+^1, Cl^-^1 and Cl^-^2), family effect was not significant (P > 0.05).

### Phenotypic correlations

As shown in Table 2, the phenotypic correlation between the values of sodium and chloride concentrations at the two challenges was high for sodium (Pearson coefficient of correlation R = 0.67) but low for chloride (R = 0.08). Within challenge, the phenotypic correlation between sodium and chloride concentrations was moderate (R = 0.21). No significant phenotypic correlation was detected between gill index and any of the plasma ion concentrations.

**Table 2:** Pairwise coefficients of phenotypic correlation among the different traits

Traits		gill index	**Cl**^**-**^**2**	**Cl**^**-**^**1**	**Na**^**+**^**2**
Na^+^1	R	0.00	0.03	**0.21**	**0.67**
	*P*	*0.98*	*0.31*	***<0.0001***	***<0.0001***
	n	776	862	901	871

Na^+^2	R	0.06	**0.22**	**0.13**	
	*P*	*0.12*	***<0.0001***	***0.0001***	
	n	771	865	867	
	
Cl^-^1	R	0.01	**0.08**		
	*P*	*0.69*	***0.02***		
	n	772	858		
		
Cl^-^2	R	0.04			
	*P*	*0.24*			
	n	762			

R: Pearson coefficient of correlation between individual records corrected for fixed effects and co-factors; *P*: associated P-value; n: number of individual records.

### QTL detection

#### Results of unitrait analyses

Unitrait analyses highlighted the existence of seven significant (P < 0.05) and seven suggestive (P < 0.10) QTL. Ten QTL affected plasma ion concentrations (six suggestive and four significant) and four affected gill index (one suggestive and three significant, Table 3).

**Table 3:** QTL detected after unitrait analyses for gill index and ion plasma concentrations 24 h after an osmotic challenge at 30 ppt salinity

Trait	**LG (Chr)**^**a**^	**Max LRT**^**b**^	**Position **^**c**^	**CI **^**d**^	**QTL effect **^**e**^		**n**_**H**_^**f**^	
					
					mean	min-max	LR	HR
Na^+^1	RT14 (Omy19)	22.1	66	60-72	0.33	0.26-0.47	4	1
	RT29 (Omy17)	23.3	55	52-60	0.34	0.22-0.55	4	1
	RT31 (Omy3)	22.8	4	0-14	0.31	0.22-0.49	4	2

Na^+^2	RT14 (Omy19)	28.9*	0	0-10	0.45	0.24-0.64	2	1
	RT19 (Omy11)	22.3	119	115-119	0.30	0.21-0.38	5	2

Cl^-^1	RT25 (Omy29)	25.1*	12	6-19	0.29	0.20-0.43	5	2
	RT26 (Omy24)	22.8*	43	27-55	0.30	0.20-0.48	6	0

Cl^-^2	RT10 (Omy6)	31.2**	21	18-24	0.41	0.21-0.83	3	4
	RT19 (Omy11)	22.7	112	88-119	0.34	0.26-0.56	4	2
	RT21 (Omy9)	21.7	32	0-44	0.47	0.29-0.57	2	1

Gill index	RT9 (Omy12)	28.6**	75	70-80	0.45	0.28-0.58	1	3
	RT15 (Omy21)	23.2*	51	40-51	0.33	0.20-0.49	1	4
	RT26 (Omy24)	24.7*	43	35-50	0.55	0.38-0.89	3	0
	RT27 (Omy2)	22.4	6	0-20	0.31	0.22-0.53	4	2

^a^: linkage groups according to Guyomard *et al. *[19] and corresponding physical chromosomes (Chr) according to Phillips *et al. *[20].

^b^: maximum likelihood test value (LRT) and corresponding chromosome-wide significance level (no indication: P < 0.10; *: P < 0.05; **: P < 0.01).

^c^: position on the consensus linkage group rebuilt with the families of the design.

^d^: confidence interval of the position estimated with the 'drop off' method.

^e^: QTL substitution effect (in phenotypic standard deviation of the trait). Mean, min and max refer respectively to the mean, minimum and maximum QTL effects recorded among families with significant QTL effect at P < 0.05.

^f^: n_H _is the number of F1 parent segregating for the QTL. LR and HR indicate the line having transmitted the allele with an increasing effect on the trait.

The four significant QTL for plasma ion concentrations were distributed on four different linkage groups (RT10, RT14, RT25 and RT26). Linkage group RT14 accommodated QTL for Na^+^2 (P < 0.05) and probably for Na^+^1 also (P < 0.10), but there was no overlap between the QTL locations, indicating that the QTL involved in the first challenge, if confirmed, may not have been the same as in the second challenge. Two QTL were associated with Cl^-^1 (RT25 and RT26), and one QTL was associated with Cl^-^2 (RT10). The QTL located on RT10 was the most significant (P < 0.01). No common QTL between the two successive challenges was detected for either Cl^- ^or Na^+ ^concentrations. Overall, QTL for sodium and chloride concentrations were localized in different regions of the genome. Yet, at the second challenge, suggestive QTL detected on RT19 for Na^+^2 and Cl^-^2 overlapped suggesting the existence of a QTL possibly affecting both traits.

Significant QTL for plasma ion concentrations segregated in at least 3 families of the design, and up to 7 heterozygous parents were detected for some QTL. Most often, the QTL allele that increased sodium or chloride concentrations originated from the LR grand-parent (16 cases out of 23 significant allelic contrasts for RT10, RT14, RT25 and RT26, see Table 3). The QTL alleles that increased gill index were equally distributed among the LR and HR genetic lineage (Table 3), though the lineage effect may differ according to the locus (increasing effect of HR alleles at RT9 and RT15 and decreasing effect on RT26 and RT27).

Most QTL for gill index were found on linkage groups different from those harbouring QTL for plasma ion concentrations, with the exception of RT26 where a QTL for Cl^-^1 overlapped the QTL for gill index. In the only parent where the comparison was possible, the same QTL allele increased both gill index and Cl^-^1 plasma concentration.

Mean QTL effects ranged from 0.29 to 0.55 phenotypic standard deviation. The largest allelic effects were identified for chloride concentrations (more than 0.8 phenotypic SD in some families).

#### Results of multiple-trait analyses

Altogether, multiple-trait analyses detected six significant QTL and eleven suggestive ones (Table 4).

**Table 4:** QTL detected after multiple-trait analyses for gill index and ion plasma concentrations 24 h after an osmotic challenge at 30 ppt salinity

Analysis	**Traits**^**a**^	**LG (Chr)**^**b**^	**Max LRT**^**c**^	**Position **^**d**^	**CI **^**e**^	**QTL effect**^**f**^					**n**_**H**_^**g**^	
						
						**Na**^**+**^**1**	**Na**^**+**^**2**	**Cl**^**-**^**1**	**Cl**^**-**^**2**	Gill index	LR	HR
**Significant QTL**												

2 traits	Cl^-^	RT4 (Omy25)	32.6 *	14	2-25			0.51	0.29		5	3
		RT10 (Omy6)	37.1 *	21	14-27			0.26	0.41		4	2
	
	Challenge 2	RT10 (Omy6)	46.5 ***	21	16-28		0.29		0.45		6	4
		RT19 (Omy11)	39.4 *	119	105-119		0.28		0.31		8	3

4 traits	Plasma ions	RT10 (Omy6)	65.4 *	20	18-28	0.27	0.43	0.26	0.41		4	9

5 traits	All traits	RT10 (Omy6)	76.8 *	26	20-37	0.25	0.31	0.23	0.33	0.32	9 (4)	6(2)

**Suggestive QTL**												

2 traits	Na^+^	RT14 (Omy19)	34.6	27	20-34	0.30	0.35				6	4
	Cl^-^	RT5 (Omy22)	33.1	30	0-34			0.36	0.29		0	7
		RT23 (Omy8)	35.8	114	103-123			0.37	0.52		5	7
		RT26 (Omy24)	32.6	55	42-55			0.35	0.26		7	1
	
	Challenge 1	RT7 (Omy15)	37.1	0	0-10	0.45		0.38			7	3
	
	Challenge 2	RT9 (Omy12)	36.1	17	8-25		0.45		0.49		3	6
		RT23 (Omy8)	36.2	108	90-128		0.41		0.53		3	8

4 traits	Plasma ions	RT5 (Omy22)	60.2	30	0-34	0.32	0.31	0.37	0.29		4	8
		RT19 (Omy11)	63.8	119	93-119	0.36	0.29	0.29	0.30		11	4

5 traits	All traits	RT4 (Omy 25)	64.9	10	0-11	0.23	0.33	0.54	0.33	0.39	13(2)	2(2)
		RT19 (Omy11)	76.5	119	83-119	0.34	0.36	0.26	0.31	0.29	13(1)	4(1)

^a: ^Challenge 1,2: multiple-trait analysis for respectively (Na^+^1,Cl^-^1) and (Na^+^2, Cl^-^2); Na^+^, Cl^-^: multiple-trait analysis for respectively (Na^+^1, Na^+^2) and (Cl^-^1, Cl^-^2); Plasma ions: multivariate analysis for (Na^+^1, Na^+^2, Cl^-^1, Cl^-^2).

^b ^: linkage group, according to Guyomard *et al. *[19] and corresponding physical chromosomes (Chr) according to Phillips *et al. *[20].

^c^: maximum likelihood test value (LRT) and corresponding chromosome-wide significance level (no indication: P < 0.10; *: P < 0.05; ***: P < 0.005); *** also indicates a genome-wide significance level at P < 0.05.

^d^: position on the consensus linkage group rebuilt with the families of the design.

^e^: confidence interval of the position estimated with the 'drop off' method.

^f^: mean QTL substitution effect (in phenotypic standard deviation of the trait) in the families with significant QTL effect (P < 0.05).

^g^: nH is the total number of F1 parent segregating for the QTL (in brackets: number of parents for gill index). LR and HR indicate the line having  transmitted the allele with an increasing effect on the trait.

For ionic concentrations, multiple-trait analyses confirmed the existence and location of several of the QTL previously detected in unitrait analysis (RT10, RT26, RT19). It evidenced that those QTL steadily affected the target trait in the two successive challenges or consistently affected both sodium and chloride concentrations. Thus, the highly significant QTL on RT10 for chloride concentration at the second challenge was confirmed at the first challenge (Cl^-^1-Cl^-^2 multiple-trait analysis, Table 4), and multiple-trait analysis also revealed an effect of this QTL on sodium concentration at both challenges (Na^+^2-Cl^-^2 and 4-traits analysis of plasma ions). In the 2-traits analysis for challenge 2, this QTL was significant at the genome-wide level (P < 0.05). Similarly, the effect of the QTL on RT19 on both chloride and sodium concentrations was underlined. While in unitrait analyses, this QTL was suggestive for the two traits at the second challenge, it became significant in the 2-traits analysis, and the 4-traits analysis also suggested it may be involved at the first challenge. The QTL for chloride concentration on RT26, which was detected in unitrait analysis for the first challenge only, was found again (P < 0.10) for both Cl^-^1 and Cl^-^2 in multiple-trait analysis. For those three QTL (RT10, RT19 and RT26), locations of the QTL revealed in the different analyses were quite consistent (Table 3). Results of multiple-trait analysis also confirmed that RT14 harbours QTL affecting sodium concentrations at both challenges. However, locations did not support the hypothesis of a single QTL for all traits (two QTL at 0 and 66 cM in unitrait analysis, and one QTL at 27 cM in multiple-trait analysis, with no overlap). Further testing for the presence of 2 QTL *vs *1 QTL on the linkage group did not support the presence of 2 distinct QTL (data not shown).

Novel QTL affecting plasma ion concentrations were discovered (RT4, RT5, RT7, RT9, RT23) though most of them, with the exception of QTL on RT4, were suggestive only (P < 0.10). Nevertheless, two of them were detected in several multiple-trait analyses at very consistent locations (RT5, RT23). All newly discovered QTL affected concentrations of both ions (sodium and chloride). Two of them were specific to the challenge (QTL on RT7 for challenge 1 and QTL on RT9 for challenge 2), but were significant at P < 0.10 only.

In summary, considering both significance levels and consistency of the different analyses, QTL for plasma ion concentrations were detected on the linkage groups RT4, RT10 and RT19 (both chloride and sodium concentrations at both challenges), RT26 (chloride concentration at both challenges), RT25 (chloride concentration at the first challenge) and RT14 (sodium concentration at both challenges) though no consistent QTL location could be found on that linkage group.

Allelic effects at the different QTL tended to be consistent for both ions, *i.e. *an allele that increased chloride concentration also increased sodium concentration, but opposite were also observed (often for an allelic effect near the significance threshold). For the three significant QTL affecting both ion concentrations (RT4, RT10, RT19), QTL allelic effects were consistent for both ions in 13 out of the 16 cases where the comparison was possible. Allelic effects on plasma ion concentrations also varied according to QTL and genetic lineage. While HR alleles tended to decrease chloride and sodium concentrations at QTL on linkage groups RT19 and RT26 as previously observed in unitrait analysis, they tended to be mostly associated with higher plasma concentrations at QTL on RT5, RT9 and RT23. Mean allelic effects were in the same range as in unitrait analyses (0.3-0.35 phenotypic SD). Again, the highest values were recorded for chloride concentrations (around 0.8 SD in some families, data not shown).

Considering the gill index, multiple-trait analyses detected three QTL (one significant and two suggestive) that were not found with unitrait analysis (5-traits analysis, Table 4). Those new QTL were also consistently involved in the control of plasma ion concentrations in both challenges (RT4, RT10 and RT19). Linkage group RT9 that was identified as the location of QTL for gill index in unitrait analysis (Table 3) was also identified as accommodating a suggestive QTL for ionic concentrations at the second challenge in the multiple-trait analysis (Table 4) but differences in relative position indicated that these were unlikely to be the same QTL.

There was no evidence of any lineage effect on the value of gill index. Moreover, there was no consistency between QTL allelic effects on gill index and on ionic concentrations, *i.e. *an allele increasing relative gill weight may be associated with either an increase or a decrease of post-challenge ionic concentrations in the plasma, depending on the parent and QTL (data not shown).

**Figure 2:**
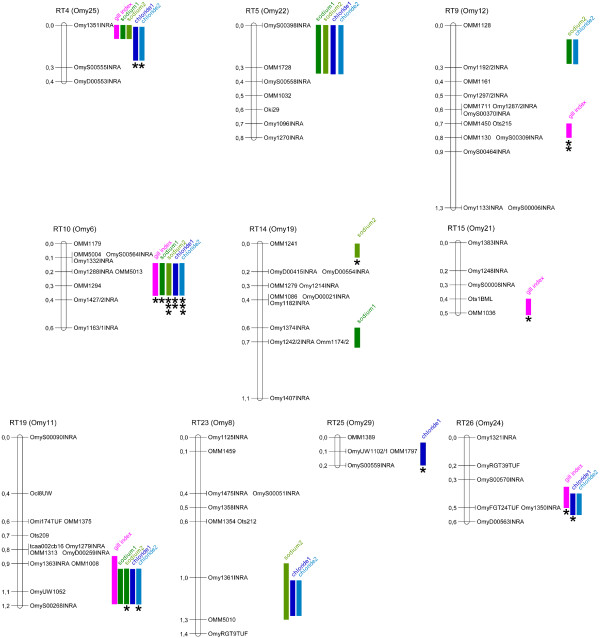
**QTL detected in the rainbow trout genome from unitrait and multiple-trait analyses for gill index and sodium and chloride plasma concentrations 24 h after osmotic challenge at 30 ppt salinity**. Only linkage groups with significant or highly suggestive QTL are shown. Linkage groups are labelled according to Guyomard *et al. *[19] with corresponding physical chromosomes (Omy) according to Phillips *et al. *[20]. Positions of markers are given on the consensus linkage groups rebuilt with the families of the design (distance in Morgans). Coloured traits indicate the presence of a QTL within the confidence interval with the name of the trait, and the highest level of significance from unitrait and multiple-trait analyses (no symbol: P < 0.10; *: P < 0.05; **: P < 0.01 at the chromosome-wide level and ***: P < 0.005 at the chromosome-wide level and P < 0.05 at the genome-wide level).

Figure 2 summarizes the major results of QTL detection. Altogether, considering both unitrait and multiple-trait analyses, six significant QTL were found for post-challenge ion concentrations in the rainbow trout genome (RT4, RT10, RT14, RT19, RT25, RT26). Two additional QTL that were consistently found in several analyses can be retained as highly suggestive (RT5, RT23). Five affected both sodium and chloride concentrations (RT4, RT5, RT10, RT19, RT23), two affected chloride concentrations only (RT25, RT26) and one affected sodium concentration only (RT14). Six QTL affecting gill index were discovered. Two were specific to the trait (RT9, RT15), while the others were also identified as QTL for post-challenge ion concentrations.

## Discussion

In this study, we have identified several genomic regions associated with the variability of response to a hyperosmotic challenge in rainbow trout. To our knowledge, this is the first description of QTL affecting plasma indicators of the hydromineral balance after an osmotic challenge in a euryhaline fish. Our results strengthen and extend previous studies which suggested that genetic factors are likely to influence the ability to adapt to high salinity in teleost fish. These studies were based on the comparison of different species and their hybrids (as in sea bass [7] or in tilapia [45]) or on the comparison of different strains or populations in teleost fish on striped bass [8], salmon [13,14,16,17,46] and artic charr (*Salvelinus alpinus*) [15]. The discovery of QTL associated with osmoregulatory ability in rainbow trout paves the way for further understanding of the genetic basis of the regulatory mechanisms and physiological pathways involved in the control of hydromineral balance in this species.

We identified several QTL contributing to the variation of plasma ion concentrations after exposure to the salinity challenge. The plasma levels of chloride and sodium, the two main ions responsible for plasma osmotic pressure, are key indicators of the regulation of hydromineral balance and consequently were chosen to monitor the adaptation to 30 ppt salinity in the QTL progenies.

Two significant QTL (on RT10 and RT19) and four suggestive QTL (on RT4, RT5, RT9 and RT23) were found to control the variation of both ions during the two successive challenges. Moreover, in most cases, the QTL affected both concentrations in the same direction. There are no data about the value of the genetic correlation between the two ion concentrations in the plasma and the genetic structure of our sample (5 full-sib families) did not allow a reliable estimate of it. Nonetheless, this result is consistent with our observation that the individual plasma values of Na^+ ^and Cl^- ^are significantly correlated within each challenge. It is also consistent with our present understanding of the hypoosmoregulatory mechanisms in seawater which indicate that the extrusion of Na^+ ^and Cl^- ^is accomplished by distinct but coupled mechanisms. The current model for NaCl extrusion at the gill epithelium proposes a basolateral co-transport of Na^+ ^and Cl^- ^down the electrochemical gradient produced by NKCC co-transporter. This co-transport is coupled with an apical extrusion of Cl^- ^via a low conductance anion channel and a paracellular extrusion of Na^+ ^through Na^+^-K^+^-ATPase transporter and diffusion towards the external medium down its electrochemical gradient (see reviews Evans et al. [4]; Hwang and Lee [5]). The morpho-functional modification of the gill epithelium characterized by the development of leaky junctions and also accessory cells are fundamental for efficient extrusion of Cl^- ^and Na^+ ^in the gill [47]. Whether these common QTL correspond to genes encoding major regulators of these cellular changes would be an interesting hypothesis to test.

We also found QTL affecting the concentration of one only of the two ions. For sodium, the most significant QTL affected ionic concentration at the second challenge (P < 0.05), and was located on RT14. For chloride, one QTL was consistently found on RT26, and an additional QTL may be located on RT25 (P < 0.05, in unitrait analysis only). Taken together, the levels of significance of those QTL remain moderate and the power of our design may have impeded the detection of some effect on the alternative ion. Nevertheless, this result may also indicate that the regulation of plasma sodium and chloride relies in part on ion-specific mechanisms. Based on the present knowledge of the cellular mechanisms responsible for Na^+ ^and Cl^- ^extrusion in hyperosmotic environment (see review Marshall and Bellamy [48]), one may hypothesise an association between QTL and one of the ion transporters characterized at the level of the gill epithelium. Additional information may have come from the genes in which the SNP used for the genome scan have been detected although examination of the known biological function of these genes (Gene Ontology) did not suggest any gene relevant for its involvement in ion or water exchange at the level of transporting epithelia.

The QTL families were F2 progenies from two divergent grand-parental lines (HR and LR) previously selected for plasma cortisol response to an acute confinement stress [33,34]. It is well established that cortisol plays an important role in the success of the adaptive osmoregulation process after transfer to a saline environment [49]. Cortisol represents a major endocrine actor in the regulation of ionic homeostasis, particularly after environmental salinity change, by regulating some seawater specific ion transporters such as Na^+^-K^+^-ATPase isoforms, NKCC and CFTR [49-56]. By its actions on gill chloride cells number and activity, cortisol acts in synergy with IGF-1 and GH to increase overall salinity tolerance (see review by McCormick [51]). Yet, altogether, our QTL results do not support evidence of a unidirectional association between the HR *vs *LR origin of QTL alleles and their effect on ion concentrations in the plasma. They rather suggest that the role of grand-parental alleles depends on the loci, a result that highlights the functional complexity of underlying mechanisms and is in agreement with the present knowledge that different stressors (as confinement or salinity stressors) have distinct effects on the physiological mechanisms regulated by cortisol [57]. However, by introducing a valuable source of additional variability in comparison to a standard within-population QTL design, the HR and LR fish provide a valuable tool with which to obtain a better understanding of the complex hormonal control of hypo-osmoregulatory mechanisms. An increasing amount of attention is paid to welfare and robustness traits in aquaculture, and many studies on the genetic control of cortisol response to stressors have been carried out, ultimately concerned with reducing stress in farmed fish [28,57,59-62]. Thus, a better understanding of the relationship between cortisol responsiveness to acute stress and osmoregulation capacities is a key requirement prior to the adoption of any selection programme designed to manage the cortisol response to stress in aquacultured fish.

Preliminary observations had suggested that LR individuals had relatively larger gill arches than HR individuals. The QTL design was thus appropriate to search for QTL for gill relative development. Four significant and two suggestive QTL were detected for gill weight relative to body weight. This result is in agreement with other studies that have identified the existence of numerous QTL for body traits in fish [62-64]. Four of those QTL are also involved in the regulation of plasma Na^+ ^and Cl^- ^concentration after the osmotic challenge, which raises the question of the possible role of gill size and morphology on osmoregulatory capacities. To our knowledge there are no published data indicating any genetic correlation between gill size and tolerance to high salinity, and the genetic structure of the QTL data set (5 full sib families) did not allow a reliable estimate to be calculated. However, we observed that the QTL alleles associated with a larger gill also affected ionic concentrations in the plasma in different directions (increase as decrease). The total weight of gill arches is not directly representative of the relative development of the gill epithelium, which is the active site for ionic and water exchange and it is therefore unlikely that there is a direct 'mechanical' effect of gill size, through an increase of the branchial ion-exchange surface. This is also in agreement with the lack of correlation between individual values for gill index and any of the plasma ion concentrations in the different challenges. The most likely hypothesis is that QTL harbour multiple syntenic genes regulating the morphological trait (gill size) and physiological traits (ionic concentrations).

Up to now, very few studies have investigated the genetic architecture of osmoregulation capacity or associated traits in salmonids. To our knowledge, only Nichols *et al. *[10] have searched for QTL associated with migratory capacities (smoltification) in trout *O. mykiss*. Using a cross between nonmigratory rainbow trout and migratory steelhead trout line, they discovered 14 genomic regions (on 14 different linkage groups) associated with smoltification-related morphological traits (growth, condition factor, body coloration, morphology). The genome scan in this study was performed using AFLP markers, but at least one microsatellite per linkage group was genotyped and can be used to establish synteny with other rainbow trout maps. Interestingly, several QTL discovered in the two studies co-localize on the same rainbow trout linkage groups. This is the case for the QTL on linkage groups RT9, RT14 and RT23 for which we could establish the synteny with respectively OC9, OC14 and OC23 in Nichols *et al*. Ambiguity remains for the QTL located respectively on OC3 in Nichols *et al*. and RT25 in this study. In Nichols *et al*., OC3 is identified by One8ASC and Ogo2. These two markers belong to a region homologous to RT25, as attested by several shared duplicated loci, including Ogo2 [19,21]. This suggests that the two aforementioned QTLs could be under control of two homeologous genes. Furthermore, since the duplicated or non-duplicated status of One8ASC (as any marker) is difficult to prove and can vary among populations, the possibility that OC3 and RT25 correspond to the same linkage group in these two particular studies cannot be ruled out. Two additional QTL suggestive in the present study (RT21 and RT31) also locate on linkage groups identified in Nichols *et al*. For RT21, the synteny can be established from Omy325DU (on OC21 in the study by Nichols *et al*.) and Omy325UoG (on RT21 in our linkage map [19]), which correspond in fact to the same marker (R. Danzmann, personal communication).

On the other hand, it is worth emphasising that in our study, we do not find any salinity acclimation QTL on RT20, the linkage group the most strongly associated with smoltification traits (mostly growth and morphological traits) in the study of Nichols *et al*. As no smoltification process occurred in the fish during our survey, this observation is not surprising. It emphasizes that the smoltification process and the adaptation to salinity after a direct transfer to seawater are two different processes.

Overall, there are at least three linkage groups (and possibly 6) harbouring QTL in both studies. It is very difficult to compare the location of the QTL discovered in the two studies on the linkage groups, as genome scans were performed with a low marker density and different types of markers were used (AFLP *vs *microsatellites). Moreover, the traits recorded in the two studies are quite different (survey of growth and changes in body colour and morphology over the time course of smoltification *vs *plasma indicators of the status of the hydromineral balance after an osmotic challenge in a nonmigratory population) and many linkage groups are found to harbour QTL for the traits examined in the two studies (10 and 14 in the present study and the one by Nichols *et al*. respectively). Although it is thus possible that common linkage groups are observed by chance, these observations may also substantiate the role of those genomic regions in the osmoregulatory process in trout and corroborates the hypothesis that some of the key processes for the control of hydromineral balance during adaptation to salinity (as highlighted by our QTL study) also occur in individuals undergoing smoltification.

During this experiment, a large number of individual fish were submitted twice to the same salinity stressor. The data set provides additional information on basic physiological responses of trout during the early (first 24 hours) adaptation to seawater. The mean plasma ion concentrations were in the same range as those usually recorded in similar tests with fish of similar sizes. However, when the fish were exposed twice to the salinity challenge (24 h at 30 ppt salinity) separated by a 2 week recovery period, plasma Na^+ ^and Cl^- ^levels did not behave similarly: we observed an increase in the mean plasma Na^+ ^levels between the first and second challenges whereas no major changes were observed in plasma Cl^- ^levels (Table 1). It is possible that this difference is linked to the fact that, in rainbow trout, plasma Na^+ ^level changes after transfer to SW were much more sensitive than plasma Cl^- ^level changes ([30]; Brard *et al*., unpublished data) and this requires further investigation. These results also suggest that, at least for Na^+^, an acute salinity stressor (24 h exposure to 30 ppt salinity) may lead to permanent modification of the functionality of the Na^+ ^osmoregulatory mechanisms resulting in a change in the capacity to excrete Na^+ ^when the fish is re-exposed to high salinity. To our knowledge, such a mechanism has never been reported in the literature.

## Conclusions

To summarize, we have identified several regions of the rainbow trout genome that are associated with the ability to regulate hydromineral balance after a salinity challenge. These are first findings that will undoubtedly be refined in the future as the current development of genomic tools and markers in rainbow trout will allow the use of a higher marker density and performance of genome-wide analyses. Nevertheless, the results highlight the complexity of physiological and hormonal mechanisms that underlie these processes and provide an original insight into the genetic bases of the early stages of adaptation to seawater. They also lay the foundation for further investigations and a better understanding of this complex trait.

## Authors' contributions

YLB and ND performed the QTL analyses. YLB carried out part of the plasma assays and drafted the manuscript. FK carried out the whole SNP subset of the genome scan and participated in the organisation of the overall genome scan and the generation of linkage maps. OF wrote severals adaptations of the program for the QTL detection in this protocole. RG participated in critically analysing genetic maps. MB assisted in the building of the maps. HB participated in the choice of the QTL family design. TPG supervised the production and management of fish, carried out the salinity challenge and sampling. PP designed the salinity test and shared the drafting of the manuscript. PLR wrote update versions of the QTLMap software, supervised QTL analyses and participated in drafting the manuscript. EQ conceived of the whole study, participated in its design and coordination and was involved in drafting the manuscript. All authors read and approved the final manuscript.

## Supplementary Material

Additional file 1 GenBank dbSNP submission numbers of the SNP used in the genome scan. Click here for file
