# Supplementary material for: Detection of QTL with effects on osmoregulation capacities in the rainbow trout (Oncorhynchus mykiss)
Source: BMC Genet. 2011 May 14;12:46. doi: 10.1186/1471-2156-12-46 (PMC3120726; doi:10.1186/1471-2156-12-46)
Supplement: Additional file 1 — GenBank dbSNP submission numbers of the SNP used in the genome scan. [file 1471-2156-12-46-S1.PDF]

local_identifier	NCBI_ss#
OmyD00021INRA	275362975
OmyD00029INRA	283950563
OmyD00082INRA	283950661
OmyD00096INRA	275362985
OmyD00173INRA	275363001
OmyD00259INRA	275363025
OmyD00306INRA	275363036
OmyD00353INRA	283950582
OmyD00371INRA	283950560
OmyD00405INRA	283950557
OmyD00415INRA	275363052
OmyD00499INRA	283950635
OmyD00553INRA	275363070
OmyD00554INRA	275363076
OmyD00563INRA	275363100
OmyD00565INRA	283950608
OmyD00567INRA	275515738
OmyD00576INRA	283950629
OmyD00578INRA	283950642
OmyS00006INRA	275362966
OmyS00008INRA	275362970
OmyS00011INRA	283950586
OmyS00013INRA	283950590
OmyS00016INRA	283950592
OmyS00020INRA	275362972
OmyS00037INRA	283950576
OmyS00038INRA	283950640
OmyS00044INRA	283950572
OmyS00049INRA	283950594
OmyS00051INRA	275362978
OmyS00078INRA	283950637
OmyS00081INRA	283950596
OmyS00090INRA	275362983
OmyS00099INRA	275362990
OmyS00118INRA	283950602
OmyS00135INRA	275362994
OmyS00160INRA	283950627
OmyS00168INRA	283950645
OmyS00172INRA	275362997
OmyS00225INRA	283950600
OmyS00238INRA	283950566
OmyS00252INRA	283950654
OmyS00254INRA	283950588
OmyS00266INRA	275363029
OmyS00268INRA	275363031

OmyS00274INRA	283950656
OmyS00309INRA	275363039
OmyS00361INRA	283950657
OmyS00370INRA	275363043
OmyS00379INRA	283950647
OmyS00387INRA	283950633
OmyS00397INRA	283950603
OmyS00398INRA	275363046
OmyS00399INRA	275363050
OmyS00401INRA	283950578
OmyS00424INRA	275363056
OmyS00426INRA	283950664
OmyS00442INRA	283950660
OmyS00458INRA	283950650
OmyS00464INRA	275363057
OmyS00470INRA	275363061
OmyS00476INRA	283950623
OmyS00477INRA	275363063
OmyS00498INRA	275363068
OmyS00550INRA	283950565
OmyS00551INRA	283950580
OmyS00555INRA	275363078
OmyS00556INRA	275363081
OmyS00557INRA	275363085
OmyS00558INRA	275363088
OmyS00559INRA	275363090
OmyS00560INRA	283950573
OmyS00561INRA	275363093
OmyS00562INRA	275363097
OmyS00564INRA	275363103
OmyS00566INRA	275363106
OmyS00568INRA	275363109
OmyS00569INRA	283950568
OmyS00570INRA	275363112
OmyS00574INRA	283950598
OmyS00575INRA	283950624
OmyS00579INRA	283950652
OmyS00580INRA	283950606
OmyS00581INRA	283950631
OmyS00582INRA	283950583
